# Hypo- and hypercapnia predict mortality in oxygen-dependent chronic obstructive pulmonary disease: a population-based prospective study

**DOI:** 10.1186/1465-9921-15-30

**Published:** 2014-03-13

**Authors:** Zainab Ahmadi, Anna Bornefalk-Hermansson, Karl A Franklin, Bengt Midgren, Magnus P Ekström

**Affiliations:** 1 Department of Medicine, Blekinge Hospital, 37185 Karlskrona, Sweden; 2 Uppsala Clinical Research Center, Uppsala University Hospital, Uppsala, Sweden; 3 Department of Surgical and Perioperative Sciences, Surgery, University Hospital of Umeå, Umeå, Sweden; 4 Department of Clinical Sciences, Lund, Division of Respiratory Medicine & Allergology, Lund University Hospital, Lund, Sweden

**Keywords:** COPD, LTOT, Mortality, PaCO_2_, Hypercapnia, Carbon dioxide, Respiratory failure, Survival

## Abstract

**Background:**

The prognostic role of the arterial blood gas tension of carbon dioxide (PaCO_2_) in severe Chronic Obstructive Pulmonary Disease (COPD) remains unknown. The aim of this study was to estimate the association between PaCO_2_ and mortality in oxygen-dependent COPD.

**Methods:**

National prospective study of patients starting long-term oxygen therapy (LTOT) for COPD in Sweden between October 1, 2005 and June 30, 2009, with all-cause mortality as endpoint. The association between PaCO_2_ while breathing air, PaCO_2_ (air), and mortality was estimated using Cox regression adjusted for age, sex, arterial blood gas tension of oxygen (PaO_2_), World Health Organization performance status, body mass index, comorbidity, and medications.

**Results:**

Of 2,249 patients included, 1,129 (50%) died during a median 1.1 years (IQR 0.6-2.0 years) of observation. No patient was lost to follow-up. PaCO_2_ (air) independently predicted adjusted mortality (p < 0.001). The association with mortality was U-shaped, with the lowest mortality at approximately PaCO_2_ (air) 6.5 kPa and increased mortality at PaCO_2_ (air) below 5.0 kPa and above 7.0 kPa.

**Conclusion:**

In oxygen-dependent COPD, PaCO_2_ (air) is an independent prognostic factor with a U-shaped association with mortality.

## Introduction

Advanced chronic obstructive pulmonary disease (COPD) is associated with high morbidity and mortality [1]. Although long-term oxygen therapy (LTOT) decreases mortality in patients with advanced COPD and chronic hypoxemia [2,3], the prognosis is poor with a mortality rate of 51% at 2 years [4]. Predictors of mortality in oxygen-dependent COPD include sex, age, body mass index (BMI), comorbidities, forced expiratory volume in one second (FEV_1_), and arterial blood gas tension of oxygen (PaO_2_) [5-14].

The prognostic role of PaCO_2_ in oxygen-dependent COPD remains unknown. PaCO_2_ while breathing air, PaCO_2_ (air), has been associated with both increased mortality [5], decreased mortality [15], and no association with mortality [4,16].

Defining the predictive role of PaCO_2_ (air) on mortality is important for several reasons. Despite the data being inconsistent, clinicians often regard hypercapnia (PaCO_2_ > 6.5 kPa) as an indicator of more severe respiratory disease, worse prognosis, and higher risk of complications from oxygen therapy including respiratory depression. High-flow oxygen given pre-hospital to patients with likely COPD exacerbation was recently associated with increased risk of acute hypercapnia and mortality [17]. Guidelines recommend titration of oxygen dose in hospital to avoid possible adverse effects when LTOT is initiated in a patient with hypercapnia [18]. Hypercapnia might indicate the presence of a concurrent hypoventilation syndrome in COPD, which is associated with shorter survival [19]. In addition, knowledge on predictors of mortality is needed as a prognostic model for patients with oxygen-dependent COPD is lacking.

The aim of this nationwide prospective study was therefore to estimate the association between PaCO_2_ (air) and mortality in oxygen-dependent COPD.

## Materials and methods

We prospectively included patients from the National Register for Long Term Oxygen Therapy (Swedevox) with physician-diagnosed COPD who started LTOT between October 1, 2005 and June 30, 2009. Swedevox is a health quality register run by the health professionals and with financial support from the government. Swedevox has a population-based coverage of approximately 85% of patients starting LTOT in Sweden since 1987 [20]. Details of the register were described in a recently published study using the same database [21]. For patients who had started LTOT more than once (n = 62), only the most recent treatment episode was included in the analysis. Exclusion criterion was a diagnosis of lung cancer before starting LTOT (baseline).

Data on comorbidity and time spent in hospital during the four-year period before baseline were obtained from the National Patient Register for in- and outpatient care [22]. Diagnoses were coded according to the ninth (before 1997) [23] and tenth revisions of the International Classification of Disease (ICD) [24]. Possible comorbid hypoventilation was defined as hypoxia with a normal alveolar-arterial oxygen tension difference, (P[A-a]O_2_), of < 3 kPa, or a prior diagnosis (ICD-9; ICD-10) which may cause hypoventilation [25]: scoliosis (737.4; M41), spinal muscular atrophy (335.1; G12), muscular dystrophy (335.2; G71), myasthenia gravis (358; G70), post-polio syndrome (138; G14), sequelae of poliomyelitis (B91.9), sequelae of respiratory tuberculosis (137; B90.9), obstructive sleep apnea (327.2; G47.3), obesity hypoventilation syndrome (278.0; E66.2), or prolonged mechanical ventilation (DG008, AG063; 93.90, 96.70).

Data on all dispensed drug prescriptions in outpatient care after July 1, 2005 were obtained from the Swedish Prescribed Drug Register [26]. Patients were followed until withdrawal of LTOT, death, or December 31, 2009, whichever came first. The primary endpoint was all-cause mortality, obtained from the Swedish Causes of Death Register.

All patients participating in the study were informed according to directives from the authorities. The study was approved by all the relevant ethics committees in Sweden, the Swedish National Board of Health and Welfare, and the Swedish Data Inspection Board.

### Statistical analysis

Baseline patient characteristics were summarized using mean with standard deviation (SD) and median with range or interquartile range (IQR) for continuous variables with normal and skewed distribution, respectively. Categorical variables were expressed as frequencies and percentages. The differences among the groups were tested with t-test for continuous variables and chi-square test for categorical variables.

Missing elements were imputed for PaO_2_ (air) (n = 289), PaCO_2_ (air) (n = 301), PaO_2_ (oxygen) (n = 210), PaCO_2_ (oxygen) (n = 213), FEV_1_ (n = 849), and body mass index (BMI) (n = 701), as previously described [21]. The model estimates were robust to the imputations. P[A-a]O_2_ was calculated using the alveolar gas equation: P[A-a]O_2_ **=** P_i_O_2_ - (PaCO_2_/R) - PaO_2_. Change in PaCO_2_ was defined as a difference between PaCO_2_ (oxygen) and PaCO_2_ (air) of 0.3 kPa or more.

Association between PaCO_2_ (air) and mortality was estimated using Cox multiple regression, adjusting for baseline age, sex, PaO_2_ (air), World Health Organization (WHO) performance status, BMI, number of cardiovascular diagnoses (cerebrovascular disease, heart failure, hypertension, ischemic heart disease, peripheral artery disease, pulmonary embolism, and other circulatory disease), treatment with oral glucocorticoids, benzodiazepines, and opioids. The model included a linear and a squared term for PaCO_2_ (air) to evaluate nonlinear associations. Higher-order terms did not improve the model with regard to diagnostics. Mortality estimates were expressed as hazard ratios (HRs) with 95% confidence intervals (CIs). Statistical significance was defined as a two-sided p-value of < 0.05.

Statistical analyses were conducted using the software packages Stata, version 12 (StataCorp LP; College Station, TX), and SAS, version 9.2 (SAS Institute, Inc., Cary, NC).

## Results

A total of 2,249 patients were included. No patient was lost to follow-up. The cohort generated 3,118 person-years at risk during a median 1.1 years (IQR, 0.6–2.0 years) of follow-up. During this time, 1,129 (50%) patients died. The main causes of death were respiratory disease (68%), cardiovascular disease (20%), and cancer (6%). LTOT was withdrawn for other reasons than death in 138 (6%) patients, mainly because of improved oxygenation.

Patient characteristics at baseline are shown in Table 1. Hypercapnia (PaCO_2_ (air) > 6.5 kPa) was present in 39% of patients. After initiating LTOT, PaCO_2_ increased in 45% of patients, decreased in 19%, and remained unchanged (difference less than 0.3 kPa) in 36%. Of patients with hypercapnia, 37% increased in PaCO_2_ after starting LTOT, whereas 30% decreased to a PaCO_2_ below 6.5 kPa.

**Table 1:** Patient characteristics at baseline

**Characteristic**	**All patients, n = 2,249**
Age, years	74.7 ± 8.2
Male/female gender	921/1328
PaO_2_ (air), kPa	6.5 ± 0.9
PaCO_2_ (air), kPa	6.3 ± 1.2
PaO_2_ (oxygen), kPa	8.7 ± 1.1
PaCO_2_ (oxygen), kPa	6.5 ± 1.3
FEV_1_^†^	0.71 (0.6 - 1.0)
Known ever to smoke, n (%)	2.106 (94)
Body mass index, n (%)	
< 18.5	280 (13)
18.5 – 24.9	1397 (62)
25 – 29.9	338 (15)
≥ 30	234 (10)
WHO performance status, n (%)	
0	132 (6)
1	881 (39)
2	714 (32)
3	292 (13)
4	31 (1)
Missing	199 (9)
Cardiovascular diagnoses, n (%)	
0	755 (34)
1	823 (37)
2	449 (20)
>2	222 (10)

Data presented as mean ± SD unless otherwise specified. Hospitalizations and diagnoses were assessed within the four-year period before the start of long-term oxygen therapy (LTOT).

^†^Median (first quartile – third quartile).

Abbreviations: FEV_1_, forced expiratory volume in one second.

Hypercapnic patients were prescribed a slightly lower median oxygen dose than normocapnic patients, 1 (IQR, 1–1.5) vs. 1.5 (IQR, 1–2) l/min (p < 0.001). In total, 29% of patients started LTOT in hospital. A slightly greater proportion of patients with hypercapnia (31%) than normocapnic patients (27%) started LTOT in hospital (p = 0.039).

### Effects on mortality

**Figure 1:**
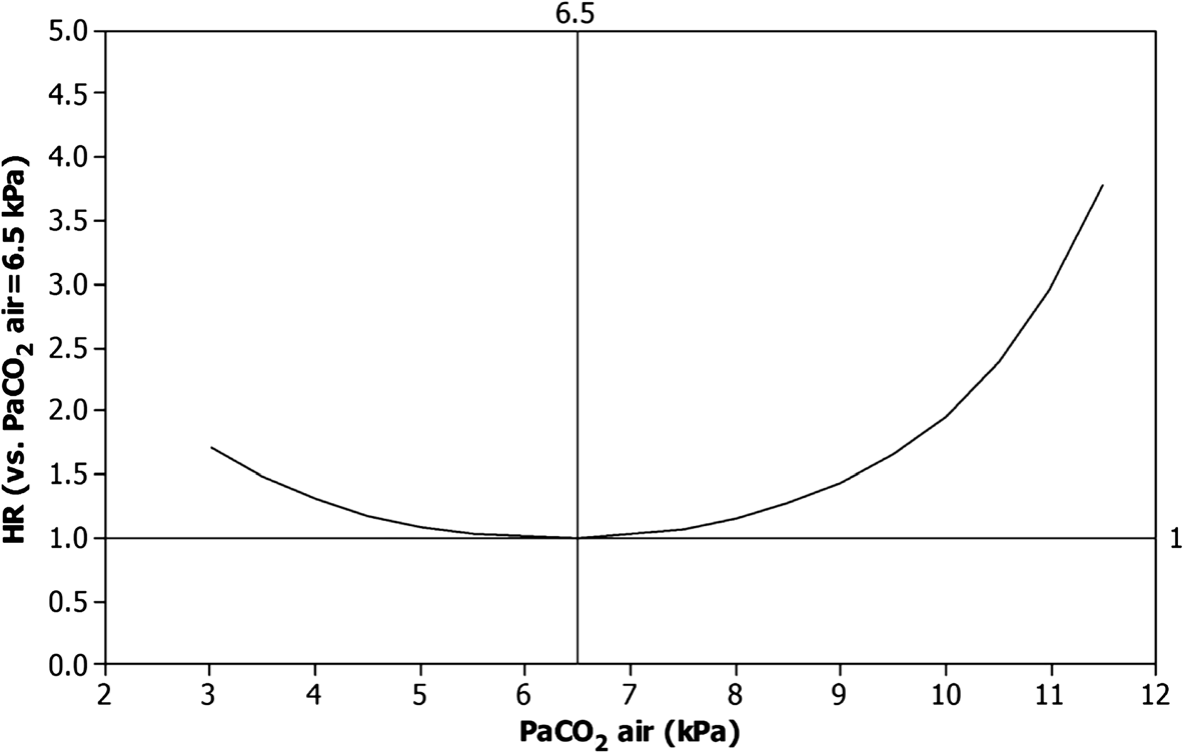
**PaCO**_**2 **_**and adjusted mortality in oxygen-dependent COPD.** Hazard ratio of death for different levels of PaCO_2_ (air) compared to at PaCO_2_ (air) = 6.5 kPa, adjusted for age, sex, PaO_2_ (air), WHO performance status, BMI, number of cardiovascular diagnoses, and treatment with oral glucocorticoids, benzodiazepines and opioids. Abbreviations: PaCO_2_ (air), Arterial blood gas tension of carbon dioxide on air; PaO_2_ (air), Arterial blood gas tension of oxygen on air; WHO, World Health Organization; BMI, Body Mass Index.

PaCO_2_ (air) independently predicted adjusted mortality (p < 0.001), as shown in Table 2. The association with mortality exhibited a U-shaped pattern, with lowest mortality at approximately PaCO_2_ (air) 6.5 kPa and increased mortality below 5.0 kPa and above 7.0 kPa (Figure 1). Compared to a PaCO_2_ (air) of 6.5 kPa, a PaCO_2_ (air) of ≤ 4.5 kPa increased mortality by 17% or more, and a PaCO_2_ (air) of ≥ 8.0 kPa increased mortality by at least 15%. Excluding patients with possible hypoventilation (n = 122) did not alter the estimates. Sensitivity analysis only including patients with FEV1/FVC < 0.7 did not alter the results.

**Table 2:** Cox regression of all-cause mortality in 2,249 patients on long-term oxygen therapy for COPD

**Parameter**	**Hazard ratio**	**95% CI**	**P-value**
PaCO_2_ (air)†	-	-	< 0.001
Age (per y)	1.04	1.03 – 1.05	< 0.001
Male	1.35	1.19 – 1.53	< 0.001
BMI			< 0.001*
< 18.5	1.35	1.14 – 1.60	< 0.001
18.5- 24.9	Ref	-	-
25 - 29.9	0.73	0.60 – 0.88	0.001
≥ 30	0.80	0.64 – 1.00	0.051
WHO performance status			< 0.001*
0	Ref	-	-
1	1.01	0.74 – 1.39	0.927
2	1.47	1.08 – 2.01	0.016
3	2.26	1.62 – 3.16	< 0.001
4	3.21	1.94 – 5.30	< 0.001
Missing	1.35	0.95 – 1.93	0.098
PaO_2_ (air) (per 1 kPa)	0.91	0.85 – 0.98	0.014
Cardiovascular diagnoses			< 0.001*
0	Ref	-	-
1	1.25	1.08 – 1.46	0.003
2	1.42	1.20 – 1.68	< 0.001
> 2	1.38	1.12 – 1.71	0.003
Oral glucocorticoids	1.16	1.02 – 1.31	< 0.001
Opioids	1.19	1.05 – 1.35	0.009
Benzodiazepines	1.18	1.03 – 1.34	0.014

*Wald test of total significance for class variables with more than two categories.

†The p-value is reported for both linear and squared term for PaCO_2_ (air).

Abbreviations: CI, confidence interval; Ref, reference category.

P[A-a]O_2_ did not predict adjusted mortality (p = 0.342), when included instead of PaO_2_ and PaCO_2_. Changes in PaCO_2_ when starting LTOT did not affect adjusted mortality; neither a rise in PaCO_2_ (p = 0.227) or a fall in PaCO_2_ (p = 0.355).

## Discussion

The novel finding in the present study is that PaCO_2_ (air) has an U-shaped association with mortality in oxygen-dependent COPD. PaCO_2_ (air) values lower than 5.0 kPa and higher than 7.0 kPa were associated with increased mortality.

Previous findings on the association between PaCO_2_ and mortality have been conflicting. Our results are consistent with studies reporting increased mortality associated with either hypocapnia [5,27] or hypercapnia [3,28,29]. In a large study of COPD patients on LTOT, Chailleux et al. found PaCO_2_ to be an independent negative predictor at 3 years follow-up [5]. However, Aida and colleagues found no association between PaCO_2_ (air) and mortality [16]. As none of the previous studies, to our knowledge, evaluated the shape of the association, the discrepancy between the findings of previous studies may partly owe to that they did not account for a nonlinear association.

Strengths of the present study include its national prospective design and that it included a large cohort of both men and women with oxygen-dependent COPD. No patient was lost to follow-up. The analysis was adjusted for relevant confounders, including comorbidity and exposure to benzodiazepines and opioids. A limitation of the study is that we lacked data on pH and base excess and could not evaluate the chronicity of the respiratory failure or the level of metabolic compensation. Nevertheless, the study presents data on the prognostic role of PaCO_2_ in patients starting LTOT for COPD that are likely to be applicable to current clinical practice.

An interesting additional finding was the large variability in the change in PaCO_2_ when starting LTOT. There is concern among clinicians that the level of PaCO_2_ will increase after starting LTOT, especially in hypercapnic patients. In the present study, 30% of patients with hypercapnia actually *decreased* their PaCO_2_ to below 6.5 kPa after initiation of LTOT and PaCO_2_ remained unchanged in 36% of patients. This may be explained by regression to the mean and by improving blood gases over time in patients starting LTOT in relation to an exacerbation. The change in PaCO_2_ between breathing room air and breathing oxygen (LTOT) did not predict mortality. This suggests that some increase in PaCO_2_ from baseline when starting LTOT is well-tolerated and not associated with worse prognosis.

Which mechanisms underpin the association between blood gas disturbances and mortality? The lack of a clear association between P[A-a]O_2_ and mortality implies that gas exchange inefficiency is not the main driver behind the increased mortality associated with hypoxemia and hypo- and hypercapnia. Our study suggests that the level of alveolar ventilation predicts mortality independently of the level of hypoxemia. Factors contributing to the increasing mortality could include more severe underlying COPD, respiratory maladaptation, and the presence of comorbidities. In hypocapnia, increased minute ventilation may cause respiratory muscle fatigue, hastened ventilatory failure and death. This might reflect a relative inability among these patients to adapt their breathing pattern to avoid respiratory muscle fatigue. Comorbidities such as congestive heart failure or pulmonary embolism may also contribute to the increased ventilation and worse prognosis. Modest hypercapnia may reflect an adaptive alveolar hypoventilation, which occurs (unconsciously) to minimize dyspnea and respiratory muscle fatigue. While modest hypercapnia were not associated with earlier death, PaCO_2_ (air) exceeding 7.0 kPa seemed to be maladaptive and associated with increased mortality rates. Mechanical defects of the chest wall, such as kyphoscoliosis, and hyperinflation may predispose to alveolar hypoventilation by imposing additional work on the inspiratory muscles [30]. Concurrent hypoventilation syndrome may contribute to hypercapnia in some COPD patients, requiring treatment with home mechanical ventilation in addition to oxygen therapy [31]. However, it is still unclear whether long-term non-invasive home ventilation improves prognosis in patients with stable hypercapnic COPD [32].

For clinicians, this study suggests that patients with PaCO_2_ (air) below 5 kPa or above 7 kPa are at increased risk of death and should be optimally treated and carefully followed. Furthermore, patients with hypo- or hypercapnia should be evaluated for comorbid diseases, including heart failure and hypoventilation syndromes. The U-shaped association of PaCO_2_ (air) with overall mortality should be validated by further studies and incorporated in future prognostic models of patients with advanced COPD.

## Conclusion

In conclusion, the level of alveolar ventilation, as reflected by both hypo- and hypercapnia, predicts mortality in oxygen-dependent COPD.

## Abbreviations

BMI: Body mass index; CI: Confidence interval; COPD: Chronic obstructive pulmonary disease; HR: Hazard ratio; ICD: International classification of disease; IQR: Interquartile range; FEV1: Forced expiratory volume in one second; LTOT: Long-term oxygen therapy; P[A-a]O2: Alveolar-arterial oxygen tension difference; PIO2: Inspired partial pressure of oxygen; R: Respiratory quotient, normally 0.8; PaO2: Arterial blood gas tension of oxygen; PaCO2: Arterial blood gas tension of carbon dioxide; PaO2 (air): Arterial blood gas tension of oxygen on air; PaO2 (oxygen): Arterial blood gas tension of oxygen on oxygen; PaCO2 (air): Arterial blood gas tension of carbon dioxide on air; PaCO2 (oxygen): Arterial blood gas tension of carbon dioxide on oxygen; Ref: Reference category; SD: Standard deviation; WHO: World health organization.

## Competing interests

The authors declare that they have no competing interest.

## Authors’ contributions

ME had full access to all the data in the study and takes full responsibility for the integrity of the data and the accuracy of the data analysis. Conception and design: ABH, ME, ZA; acquisition of data: BM, ME; analysis and interpretation of data: ABH, BM, KF, ME, ZA; drafting the article: ABH, ME, ZA; revision for important intellectual content and approval of the version to be published: ABH, BM, KF, ME, ZA. All authors read and approved the final manuscript.
